# The catalytic nature of protein aggregation

**DOI:** 10.1063/1.5133635

**Published:** 2020-01-28

**Authors:** Alexander J. Dear, Georg Meisl, Thomas C. T. Michaels, Manuela R. Zimmermann, Sara Linse, Tuomas P. J. Knowles

**Affiliations:** 1 Department of Chemistry, University of Cambridge, Lensfield Road, Cambridge CB2 1EW, United Kingdom; 2 Department of Biochemistry and Structural Biology, Lund University, SE22100 Lund, Sweden; 3 Cavendish Laboratory, University of Cambridge, J J Thomson Avenue, Cambridge CB3 0HE, United Kingdom

## Abstract

The formation of amyloid fibrils from soluble peptide is a hallmark of many neurodegenerative diseases such as Alzheimer’s and Parkinson’s diseases. Characterization of the microscopic reaction processes that underlie these phenomena have yielded insights into the progression of such diseases and may inform rational approaches for the design of drugs to halt them. Experimental evidence suggests that most of these reaction processes are intrinsically catalytic in nature and may display enzymelike saturation effects under conditions typical of biological systems, yet a unified modeling framework accounting for these saturation effects is still lacking. In this paper, we therefore present a universal kinetic model for biofilament formation in which every fundamental process in the reaction network can be catalytic. The single closed-form expression derived is capable of describing with high accuracy a wide range of mechanisms of biofilament formation and providing the first integrated rate law of a system in which multiple reaction processes are saturated. Moreover, its unprecedented mathematical simplicity permits us to very clearly interpret the effects of increasing saturation on the overall kinetics. The effectiveness of the model is illustrated by fitting it to the data of *in vitro* Aβ40 aggregation. Remarkably, we find that primary nucleation becomes saturated, demonstrating that it must be heterogeneous, occurring at interfaces and not in solution.

## I. INTRODUCTION

The self-assembly of proteins into amyloid fibrils is a natural biological process that has received increasing attention in recent years due to its close association with a range of widely prevalent, incurable, and fatal human disorders such as Alzheimer’s and Parkinson’s diseases.^1^ Identifying the reaction processes that lead to biofilament proliferation is vital for understanding these diseases. It also holds the key to the rational design of drugs to inhibit these self-assembly phenomena, with a view to halting or preventing the related disorders.

Biofilament assembly from monomeric samples is always initiated by a slow nucleation reaction process in which monomeric protein molecules associate to form new filamentous structures and continued by a fast elongation process, which ensures that the average filament grows to macroscopic lengths.^2,3^ Secondary reaction processes that frequently accompany these include filament breakage (often associated with prions^4^), filament branching (seen in, e.g., actin polymerization), and secondary nucleation (nucleation of new filaments on the surface of existing filaments, first observed with sickle hemoglobin polymerization^5–7^). Most of these processes are multistep reactions. Both theoretical and experimental considerations suggest that elongation is properly described as a two-step, catalytic process controlled by a Michaelis-Menten-like rate law.^8,9^ It has similarly been shown that secondary nucleation is catalytic and can be well-described by a similar rate law in the case of the aggregation of the Alzheimer’s-associated Aβ40 protein.^10^ Closed sets of rate laws describing the kinetics of filamentous self-assembly via every possible combination of these reaction processes may be written down by considering only the monomer concentration alongside the number and mass concentrations of filaments, rather than the full size distribution.^11–15^ Developing analytical solutions to these rate laws, and subsequently testing them against experimental data, has permitted the key reaction processes responsible for the proliferation of a range of biofilaments of interest to be determined in the past decade.^16,17^ Most notably, it has revealed that secondary nucleation is crucial to the aggregation of the Alzheimer’s-associated Aβ42 protein.^18^ In these models, it has always been assumed that concentrations are low enough that enzymelike saturation effects are rare and that the sampled range of concentrations is small enough for them to occur in at most one reaction process at a time so that the remaining processes can be accurately approximated by single-step mass-action-like rate laws. Moreover, primary nucleation is often assumed to be homogeneous and occurring in bulk such that no catalytic saturation effects are possible. However, as larger ranges of monomer concentrations are measured with increasingly accurate kinetic experiments, these assumptions no longer hold. Thus, in order to extend our descriptions of amyloid filament formation at all concentrations of interest, there is a need to allow for all processes in the kinetic framework to be explicitly catalytic and have the potential to saturate in a given experiment. Previous integrated rate laws were unable to address such concurrent saturation of several processes.

In this paper, we develop a single, fully general approximate analytical solution to the kinetics of catalytic protein aggregation. We demonstrate its accuracy to be superior to preexisting models for protein aggregation in which at most one reaction process is multistep. We next use the model to explain exactly how the overall kinetics are affected by saturation effects in each reaction process. We finally demonstrate the utility of the model by applying it to the analysis of experimental kinetic data on *in vitro* Aβ40 aggregation, finding that in addition to secondary nucleation, primary nucleation also saturates in this system.

## II. RESULTS

### A. Aggregation reaction processes can be described by Michaelis-Menten-like equations

**FIG. 1.**
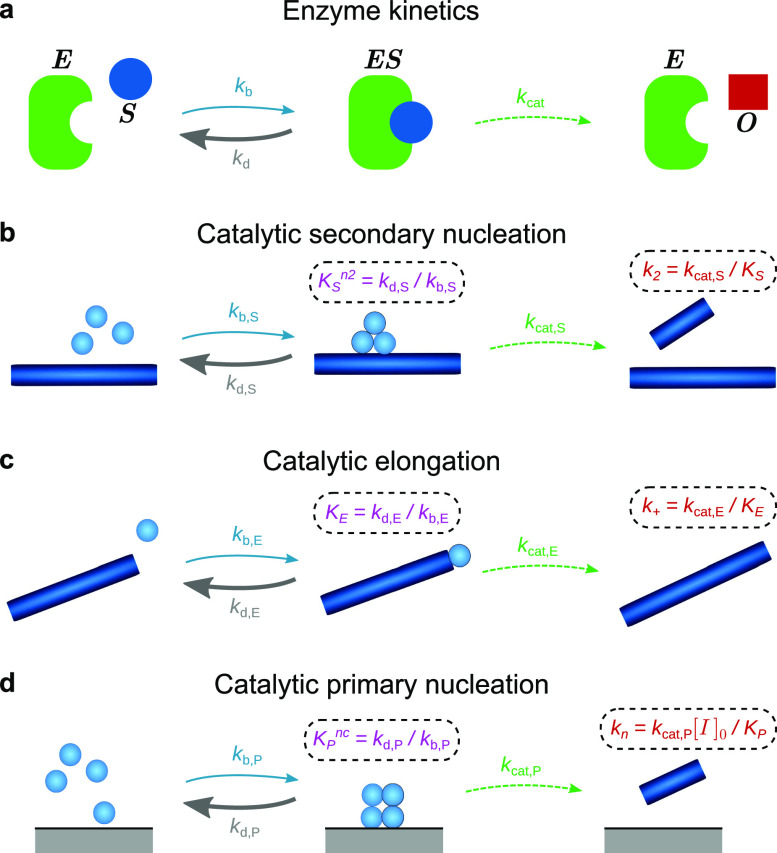
The catalytic nature of key reaction processes in biofilament assembly. (a) Catalytic conversion of substrate *S* to product *O* by an enzyme *E*, featuring an intermediate enzyme-substrate complex. (b) In secondary nucleation, the fibril surface acts as a catalyst. (c) In elongation, the growing fibril ends act as a catalyst. Although the chemical species (a shorter fibril) is not regenerated, the pseudospecies (the fibril end) is. (d) In heterogeneous primary nucleation, any surface or interface (we denote the total concentration of binding sites on the interface as ${\left[I\right]}_{0}$) present in the reaction vessel may act as a catalyst. In all cases, where the concentration is high enough, the surface may become completely saturated with monomers; at this point, further increases in concentration do not affect the rate, which is then given simply by *k*_cat_ · [*catalyst*]_*t*=0_. The 50% binding concentration *K*_*x*_ (x=P,E,S) is given by setting the intermediate bound state to steady-state, and in the usual case that $k_{d}\gg k_{cat},\;K_{x}^{n_{x}}$ is approximately the dissociation constant for the corresponding dissociation reaction. We may thus interpret *K*_*x*_ as the geometric mean of the dissociation constants for each fundamental step in the dissociation reaction.

To understand how enzymelike saturation effects arise in protein aggregation, we turn to Michaelis-Menten kinetics. This is a well-known model that describes the conversion of a substrate to a product through the catalytic action of an enzyme and admits an analytical solution.^19^ The fundamental assumptions behind this model are that the reaction is two-step, with a reversible bimolecular substrate-enzyme binding first step, followed by an irreversible unimolecular second step that regenerates the enzyme and generates the product [Fig. 1(a)]. The rate equations describing this reaction are^19^

$$\frac{d\left[ES\right]}{dt}=k_{b}\left[E\right]\left[S\right]-\left(k_{d}+k_{cat}\right)\left[ES\right],$$ (1)

$$\frac{d\left[O\right]}{dt}=k_{cat}\left[ES\right],$$ (2)

$$\left[ES\right]+\left[E\right]={\left[E\right]}_{0},$$ (3)

where [*E*], [*S*], [*ES*], [*O*], and [*E*]_0_ are the concentrations of enzyme, substrate, enzyme-bound substrate, product, and the total concentration of enzyme molecules overall.

The key additional assumption that permits an analytical solution to be determined is that the enzyme-substrate complex is either at quasi-steady-state (QSS) or at pre-equilibrium with the free substrate (PE). If this is satisfied, then the rate of reaction *r* is given by^19^

$$r=\frac{v_{max}\left[S\right]}{K_{M}+\left[S\right]},$$ (4)

where $v_{\text{max}}$ = *k*_cat_[*E*]_0_ is the maximum rate achievable by the system and the Michaelis constant *K*_*M*_ is the substrate concentration at which the rate is half of $v_{\text{max}}$.

Almost all of these assumptions hold for the catalytic reaction processes underlying biofilament formation, when the substrate is monomeric protein, and the “enzyme” is a filament surface, a growing end, or another interface in the reaction vessel [see Table I and Figs. 1(b)–1(d)]. The only assumption not generally satisfied is that the first step is bimolecular, since multiple monomers may be involved in primary and secondary nucleation. However, the mathematics are easily generalized for higher reaction orders with respect to monomers, yielding instead a Hill equation for *r*, which resembles the Michaelis-Menten equation (4) but has the monomeric substrate raised to the power of the new reaction order.

**TABLE I.** Criteria for Michaelis-Menten type kinetics are only fully satisfied by the elongation reaction. Secondary nucleation does not satisfy assumption 7 and is therefore described by the closely related Hill type kinetics instead. Primary nucleation only satisfies assumptions 4 and 5 when heterogeneous and not homogeneous. Assumption 6 is generally satisfied when the concentration of the catalyst-bound substrate is low. This may not be the case for heterogeneous primary nucleation in general, but it is for Aβ40; if the monomer-bound catalyst was present at a significant concentration relative to monomers, an extended slow increase in the fibril mass concentration toward the end of the reaction would be visible, as remaining monomers detach from the interface and attach to the ends of growing fibrils. This effect is not seen in the aggregation kinetics of Aβ40 suggesting that the concentration of surface-bound monomers is low. Assumption 6 is also satisfied for elongation due to the comparatively low concentration of growing filament ends, and for secondary nucleation because at the early times at which secondary nucleation plays a significant role, the fibril mass concentration is still much lower than the monomer concentration.

Criterion for Michalis-Menten kinetics	1° nucleation	Elongation	2° nucleation
1: Reversible initial step	*✓*	*✓*	*✓*
2: Irreversible final step	*✓*	*✓*	*✓*
3: Unimolecular final step	*✓*	*✓*	*✓*
4: Substrate-catalyst binding in initial step	(*✓*)	*✓*	*✓*
5: Final step regenerates catalyst	(*✓*)	*✓*	*✓*
6: Quasi steady-state bound catalyst	(*✓*)	*✓*	*✓*
7: Substrate-catalyst binding is bimolecular		*✓*	

For instance, secondary nucleation has been shown to be well-described by the Michaelis-Menten style rate law,^10^

$$r_{S}=\frac{k_{2}m{\left(t\right)}^{n_{2}}}{1+{\left(\frac{m\left(t\right)}{K_{S}}\right)}^{n_{2}}}M\left(t\right),$$ (5)

where *m*(*t*) is the concentration of monomeric protein at time *t*, *M*(*t*) is the mass concentration of fibrils (proportional to the concentration of fibril surface), *K*_*S*_ is the monomer concentration at which for a given *M*(*t*), the rate is equal to half the maximum possible rate $k_{2}K_{S}^{n_{2}}M\left(t\right)$, and *k*_2_ and *n*_2_ are the rate constant and the reaction order of secondary nucleation at low monomer concentrations, respectively. Note that secondary nucleation has alternatively been described as the bimolecular binding of monomers to fibril surfaces, which then are free to diffuse and to react with one another to form fibrils.^20^ However, the resultant kinetic curves for biofilament assembly too closely resemble those generated by the Michaelis-Menten style model to be distinguished by data fitting, so we use only the Michaelis-Menten style model here.

Filament elongation has previously been modeled as a dock-lock mechanism^21–23^ and as a diffusive barrier-crossing reaction.^8,9^ Although there is no chemical species that is regenerated during an elongation reaction, the number of free fibril ends [concentration 2*P*(*t*)] is recovered unchanged after temporarily decreasing, while a rearranging monomer is bound to the end. Therefore, we may consider the fibril ends as a catalytic pseudospecies^24^ playing the role of the enzyme *E* in Eq. (1). By identifying *k*_*b*_ from Eq. (1) as the rate constant for diffusion-limited attachment of the monomer to the fibril end, *k*_*d*_ as the detachment time, and *k*_cat_ as the rearrangement time for a bound monomer to form a new fibril subunit, a Michaelis-Menten rate law identical in the functional form to those proposed in Refs. 8 and 9 may then be obtained,^24^

$$r_{E}=\frac{2k_{+}m\left(t\right)}{1+\frac{m\left(t\right)}{K_{E}}}P\left(t\right),$$ (6)

where *k*_+_ is the rate of elongation at low monomer concentrations and *K*_*E*_ is the monomer concentration at which the rate is 50% of the theoretical maximum for a given concentration *P*(*t*). Note that both *K*_*E*_ and *K*_*S*_ can also be interpreted as approximate equilibrium dissociation constants of monomers from fibril ends and fibril surfaces, respectively.

Finally, if primary nucleation is heterogeneous, then it too can be expected to be catalytic and to obey a Michaelis-Menten-style law of the form

$$r_{P}=\frac{k_{n}m{\left(t\right)}^{n_{c}}}{1+{\left(\frac{m\left(t\right)}{K_{P}}\right)}^{n_{c}}},$$ (7)

where *k*_*n*_ and *n*_*c*_ are the rate constant and the reaction order of primary nucleation at low monomer concentrations, respectively, and *K*_*P*_ is the equilibrium dissociation constant of the monomer to nucleation surface and also the monomer concentration at which primary nucleation is 50% saturated.

### B. Rate equations for catalytic filamentous self-assembly

The classical single-step moment equations describing the kinetics of filament number and mass concentrations^11,12,16^ can therefore be generalized for catalytic aggregation to read

$$\frac{dP}{dt}=\frac{k_{n}m{\left(t\right)}^{n_{c}}}{1+{\left(\frac{m\left(t\right)}{K_{P}}\right)}^{n_{c}}}+\frac{k_{2}m{\left(t\right)}^{n_{2}}}{1+{\left(\frac{m\left(t\right)}{K_{S}}\right)}^{n_{2}}}M\left(t\right),$$ (8a)

$$\frac{dM}{dt}=\frac{2k_{+}m\left(t\right)}{1+\frac{m\left(t\right)}{K_{E}}}P\left(t\right).$$ (8b)

As usual, the small contributions of nucleation processes to the rate of increase in filament mass concentration have been neglected,^26^ as have contributions from processes such as filament annealing.^27^ Also note that fragmentation, instead of secondary nucleation, may be captured in these equations by setting *n*_2_ = 0. These equations can therefore describe a wide variety of filament self-assembly reactions.

### C. Analytical solutions to the kinetics of catalytic filamentous self-assembly

To solve Eqs. (8), we first nondimensionalize them and combine them into a single equation (Appendix A), yielding

$$\begin{array}{ccc} & & -\left[\frac{1}{K_{E}}\frac{d^{2}\mu \left(\tau \right)}{d\tau^{2}}+\frac{d^{2}\log\mu \left(\tau \right)}{d\tau^{2}}\right]\\ & & \;=\frac{\lambda^{2}}{\kappa^{2}}\frac{\mu {\left(\tau \right)}^{n_{c}}}{1+\mu {\left(\tau \right)}^{n_{c}}/K_{P}^{n_{c}}}+\frac{\mu {\left(\tau \right)}^{n_{2}}\left[\right.1-\mu \left(\tau \right)]}{1+\mu {\left(\tau \right)}^{n_{2}}/K_{S}^{n_{2}}}, \end{array}$$ (9)

where $\lambda =\sqrt{2k_{+}k_{n}m_{tot}^{n_{c}}}$ and $\kappa =\sqrt{2k_{+}k_{2}m_{tot}^{n_{2}+1}}$ are the effective noncatalytic fibril proliferation rates through primary and secondary processes, respectively;^28^ *μ*(*τ*) = *m*(*τ*)/*m*_tot_ is the nondimensionalized monomer concentration; and $K_{P},\;K_{E}$, and $K_{S}$ are the nondimensional dissociation constants *K*_*P*_/*m*_tot_, *K*_*E*_/*m*_tot_, and *K*_*S*_/*m*_tot_, respectively. Finally, the rate constant *k*_+_ now affects the kinetics solely via the nondimensionalized time *τ* = *κt*.

In most systems, the formation of new filaments is dominated by secondary processes, suggesting that a productive approach might be to seek a perturbation series solution in *ε* = *λ*^2^/2*κ*^2^. The resultant perturbative solution describes the early-time dynamics of Eqs. (8) as exponential growth. However, this formula is not accurate at long times as it does not account for monomer depletion and therefore does not converge. To remove this divergence and develop a global solution, we employ a perturbative renormalization group methodology similar to that used in Ref. 29 (see Appendix B for details). The resulting closed-form solution is

$$\frac{M\left(t\right)}{m_{tot}}=1-{\left[1+\frac{\varepsilon^{\prime }}{c^{\prime }}\left(e^{\kappa^{\prime }t}+e^{-\kappa^{\prime }t}-2\right)\right]}^{-c^{\prime }},where$$ (10a)

$$\varepsilon^{\prime }=\frac{k_{n}^{\prime }m_{tot}^{n_{c}}}{2k_{2}^{\prime }m_{tot}^{n_{2}+1}},$$ (10b)

$$\kappa^{\prime }=\sqrt{2k_{+}^{\prime }k_{2}^{\prime }m_{tot}^{n_{2}+1}},$$ (10c)

$$c^{\prime }=\frac{3}{2n_{2}^{\prime }+1},$$ (10d)

and *ε*′ can be interpreted as the relative importance of primary vs secondary nucleation as a source of new fibrils. $k_{n}^{\prime },\;k_{+}^{\prime }$, and $k_{2}^{\prime }$ are the perturbed rate constants for primary nucleation, elongation, and secondary nucleation, respectively, given by

$$k_{n}^{\prime }=k_{n}\frac{K_{P}^{n_{c}}}{1+K_{P}^{n_{c}}},$$ (11a)

$$k_{+}^{\prime }=k_{+}\frac{K_{E}}{1+K_{E}},$$ (11b)

$$k_{2}^{\prime }=k_{2}\frac{K_{S}^{n_{2}}}{1+K_{S}^{n_{2}}}.$$ (11c)

Note that these are simply the effective rate constants at *t* = 0, as shown by comparison with Eqs. (5)–(7). Finally, $n_{2}^{\prime }$ + 1 is the effective reaction order of filament proliferation, which also depends on the monomer concentration, given by

$$n_{2}^{\prime }=n_{2}\frac{K_{S}^{n_{2}}}{1+K_{S}^{n_{2}}}-\frac{2}{1+K_{E}}.$$ (12)

In the limit that $K_{P},\;K_{E}$, and $K_{S}$ tend to infinity (i.e., when initial monomer concentrations are far below the saturation concentrations), $k_{n}^{\prime }\to k_{n},\;k_{+}^{\prime }\to k_{+},\;k_{2}^{\prime }\to k_{2}$, and $n_{2}^{\prime }$ → *n*_2_, and single-step kinetics are recovered as required.

Previously, a range of different approximate solutions have been developed for the kinetics of biofilament formation in which at most one of the processes displays saturation effects. Comparison of these legacy models to the solution given by Eqs. (10) in Appendix C demonstrates that in almost all cases when reactions from initially monomeric samples are considered, our new solution describes the kinetics with improved accuracy. Moreover, it has a far simpler mathematical form than most of these earlier models (see Table II). In Appendix D, we further generalize the solution so that it accurately describes the kinetics of biofilament formation even when secondary processes no longer dominate over primary processes.

**TABLE II.** Performance compared to pre-existing models for biofilament assembly featuring primary nucleation, elongation, and secondary processes. *✓* indicates improvement and ≈ indicates little difference. No models have previously been derived for biofilament assembly via a catalytic primary nucleation process, but earlier models do exist in which either elongation or secondary nucleation is catalytic. In every case, it is found that the general model derived in this paper is superior to these previous specialized models, either in accuracy or in mathematical simplicity (or in both). The most notable improvement is in our description of biofilament formation with secondary nucleation and catalytic elongation for which the previous model gave a comparatively poor description. See Appendix C for full details. Note that many other combinations of reaction processes have never previously been modeled but can also be accurately described by our new approach.

Earlier model	Reaction steps	Improvement in accuracy?	Improvement in model simplicity?
Reference 11	1° nucleation; elongation; fragmentation	*✓*	≈
Reference 13	1° nucleation; elongation; 2° nucleation	≈	*✓*
Reference 25	1° nucleation; elongation; 2° nucleation	*✓*	≈
Reference 16	1° nucleation; catalytic elongation; fragmentation	≈	*✓*
Reference 16	1° nucleation; catalytic elongation; 2° nucleation	*✓ ✓*	*✓*
Reference 10	1° nucleation; elongation; catalytic 2° nucleation	*✓*	*✓*

### D. The effect of saturation on filamentous growth kinetics

The simple structure of the analytical model derived here, and its general nature, makes it particularly easy to determine the effect of saturation in different processes on the overall kinetics. From Eqs. (10), we see that the most significant effect of saturation in any process is to reduce its effective rate constant according to Eqs. (11). Saturation in elongation or in secondary nucleation also has the subsidiary effect of reducing the effective monomer dependence of the self-assembly process, by reducing $n_{2}^{\prime }$. In addition to affecting the relative difference between reactions performed at different initial monomer concentrations, this also governs how the filament mass concentration approaches its maximal value toward the end of the aggregation reaction, as the monomer is heavily depleted. Primary nucleation saturation does not affect this, as in typical small-*ε*′ systems, secondary nucleation dominates and primary nucleation ceases to affect the kinetics relatively early in the reaction.^30^

**FIG. 2.**
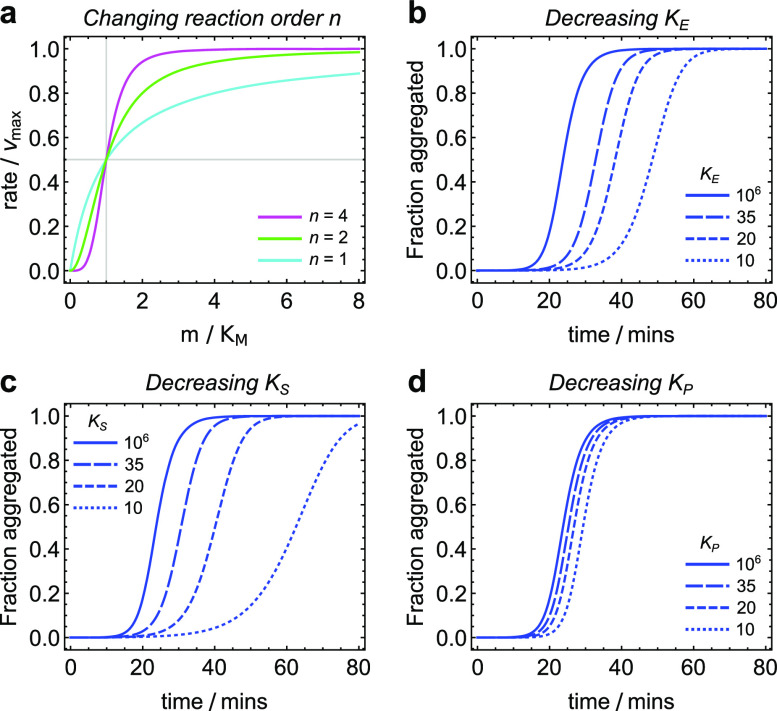
(a) Plot of the rate, scaled by the maximal rate $v_{\text{max}}$, vs the monomer concentration *m*, scaled by the half-saturation concentration *K*_*M*_, for an elongation reaction (cyan) and for nucleation reactions with orders 2 (green) and 4 (magenta). Elongation obeys Michaelis-Menten kinetics precisely, with a sublinear dependence of the rate on monomer concentration, whereas the higher-order nucleation reactions obey Hill kinetics with the rate exhibiting a sigmoidal monomer dependence. (b)–(d) investigating the effect of saturation in elongation, secondary nucleation, and primary nucleation, respectively, on aggregation curves. Aβ40 rate constants employed with *m*(0) = 35 *µ*M. Solid lines: *K*_*M*_ = 1M, i.e., no saturation. Dashed lines: *K*_*M*_ = 35, 20, or 10 *µ*M. Shorter dashed lines correspond to lower saturation concentrations. Saturation in elongation and secondary nucleation mainly reduces the aggregation rate, whereas the sole effect of saturation in primary nucleation is to increase the lag time. Due to the logarithmic dependence of the half time on primary nucleation,^26^ saturation in the latter has the smallest effect on aggregation kinetics. Saturation in secondary nucleation has the largest overall impact, despite increasing *ε*, due to the higher reaction order of secondary nucleation compared to elongation.

It has been shown elsewhere^31^ that *κ* may be interpreted as the effective first order rate constant for the proliferation of mature fibrils via secondary processes under constant-monomer conditions; *κ*′ inherits this interpretation. The kinetics are most sensitive to *κ*′ due to the exponential dependence of *M*(*t*) on *κ*′; saturation of secondary nucleation or of elongation therefore has a stronger effect on the kinetics than the saturation of primary nucleation [Figs. 2(b)–2(d)], which does not enter *κ*′. Increasing saturation in these processes dilates the reaction time, or stretches the kinetic curve on the *t*-axis, by reducing *κ*′.

Saturation of elongation does not affect the parameter *ε*′, whereas saturation of secondary nucleation increases *ε*′, which counteracts the reduction in *κ*′ to a certain extent. However, the kinetics are typically more sensitive to saturation in secondary nucleation overall [Figs. 2(b) and 2(c)], due to the higher reaction order of secondary nucleation compared to elongation [Fig. 2(a)]. *ε*′ gives the relative rate of primary nucleation compared to secondary processes and is typically expected to be small in most amyloid-forming systems. It may also be interpreted as the logarithm of an effective starting time; the only effect of increasing saturation in primary nucleation on the kinetics is therefore to shift the concentration curve to the right [Fig. 2(d)].

At concentrations far below the saturation concentration for a given process, its effective rate constant is unchanged and loses its dependence on the saturation concentration, which can therefore not be determined with any accuracy through kinetic model fitting. Far above the saturation concentration, by contrast, the effective constant for process (*x* = P,E,S) becomes $k_{x}K_{x}^{n_{x}}$, and neither the rate constant nor the saturation concentration may be determined with any accuracy but only their product, the conversion rate constant *k*_cat,*x*_ (multiplied by the nucleation site concentration [*I*]_0_ in the case of primary nucleation). This follows since the effective rate constants enter the model as $k_{n}^{\prime }m_{tot}^{n_{c}},\;k_{+}^{\prime }m_{tot}$, and $k_{2}^{\prime }m_{tot}^{n_{2}}$, which reduce in this limit to $k_{n}K_{P}^{n_{c}}=k_{cat,P}{\left[I\right]}_{0},\;k_{+}K_{E}=k_{cat,E}$, and $k_{2}K_{S}^{n_{2}}=k_{cat,S}$. Modulo factors of *M*(*t*) and *P*(*t*) for secondary nucleation and elongation, respectively, these are also the maximum rates $v_{\text{max},x}$ attainable by each reaction process. Thus, depending on the monomer concentrations spanned by the data to which the model is fitted, for a given reaction process, it may be appropriate to report both the saturation concentration and the rate constant (if the concentrations span the saturating region), or just the rate constant (if the concentrations measured are well below the saturation concentration), or just $v_{\text{max}}$ (if the concentrations measured are well above the saturation concentration).

### E. Aβ40 undergoes heterogeneous primary nucleation

**FIG. 3.**
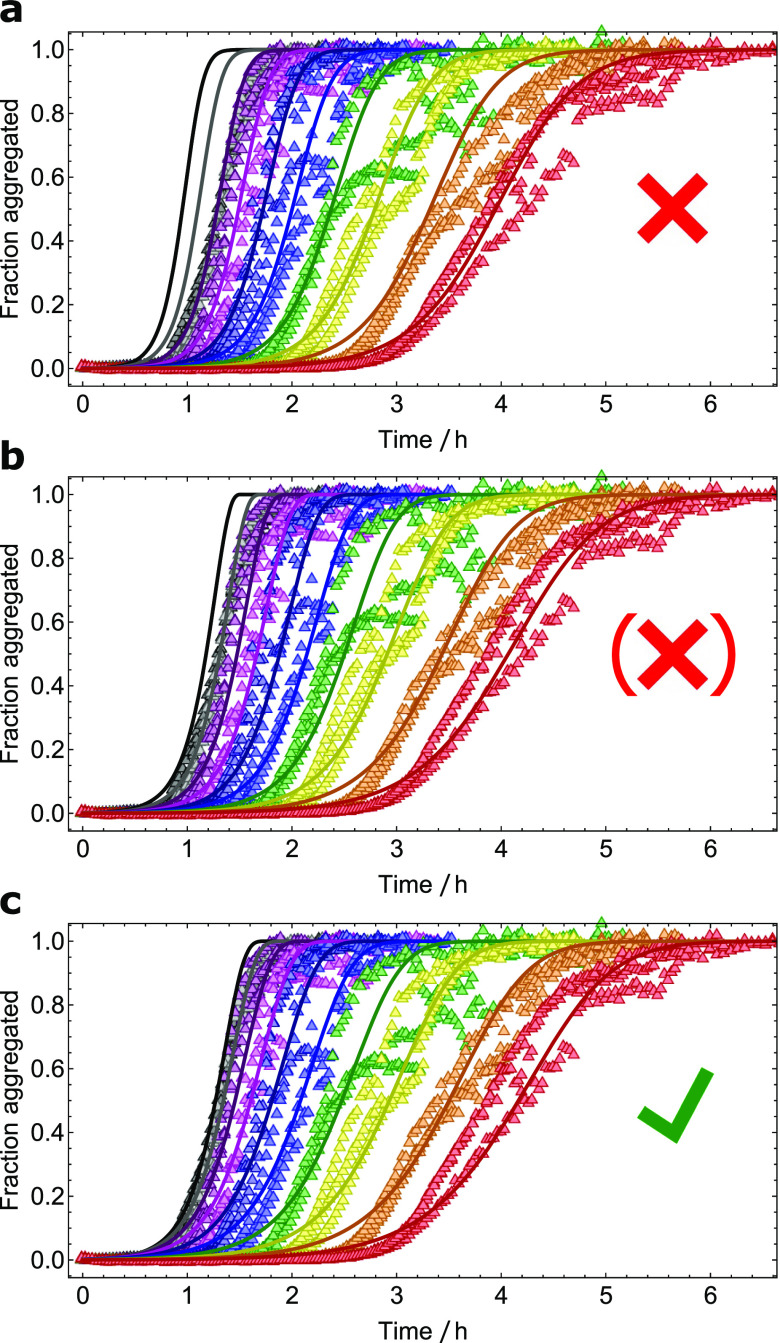
Aggregation of Aβ40 monomers into filaments; fractional aggregation monitored by ThT fluorescence vs time. Initial monomer concentrations ranging from 3.5 *µ*M to 70 *µ*M (only 9.1–70 *µ*M shown here). Data taken from Ref. 10. (a) The fit to the model by Meisl *et al.*^10^ is only able to reproduce aggregation curves below 35 *µ*M. (b) Equation (10a) with *K*_*P*_ set to an arbitrarily large value fits the data significantly better, yielding *K*_*E*_ = 103 *µ*M. (c) When fully unconstrained, Eq. (10a) fits the data better still, yielding a larger value of *K*_*E*_ and a moderate value of *K*_*P*_, suggesting that saturation in primary nucleation is more important than in elongation for this system. However, the improvement in fit quality is too small to reach a firm conclusion and more analysis is needed.

**FIG. 4.**
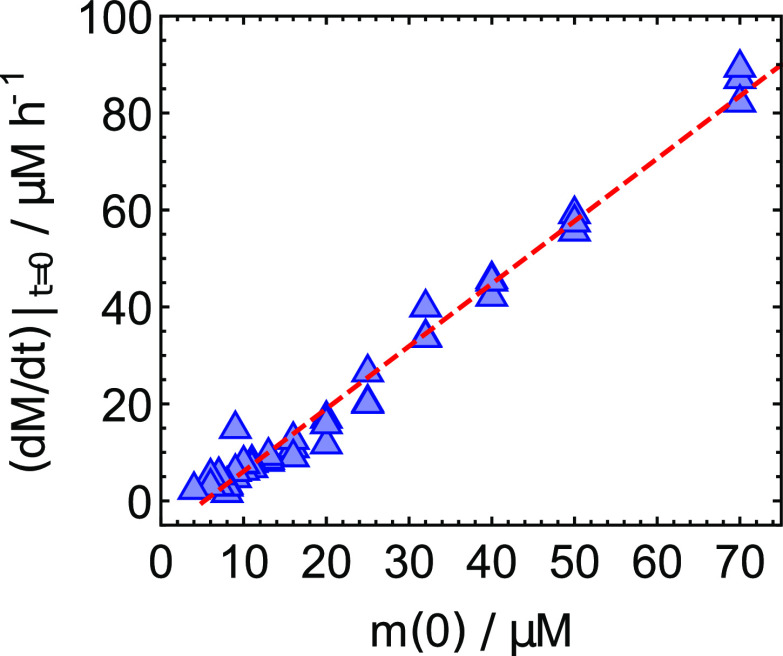
Initial rates of aggregation of Aβ40 monomers into filaments in the presence of 21 *µ*M seeds; initial monomer concentrations ranging from 3.5 *µ*M to 70 *µ*M. Data taken from Ref. 10. The initial rates in the presence of such high seeds should depend only on the elongation rate; if saturation effects are present in the elongation reaction, the initial aggregation should have a sublinear dependence on initial monomer concentration [see the cyan curve in Fig. 2(a)]. Instead, an approximately linear dependence on the monomer concentration is observed, demonstrating the absence of significant saturation in elongation at the monomer concentrations studied. This supports the tentative conclusion from Fig. 3 that the saturation effects additional to those from secondary nucleation, visible at the upper end of the concentration range studied, originate from primary nucleation and not from elongation.

**FIG. 5.**
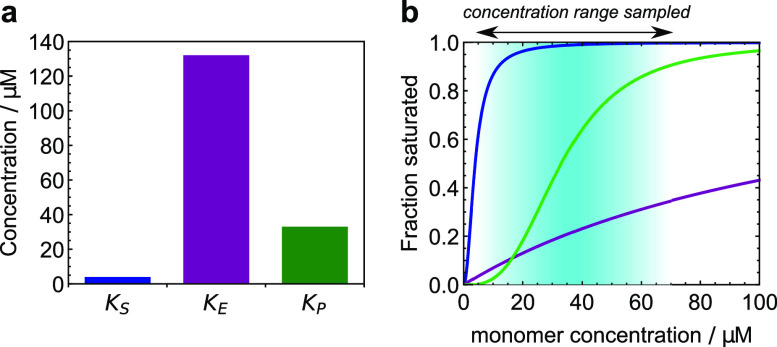
The degree of saturation of the reaction processes of Aβ40 kinetics: blue for secondary nucleation; purple for elongation, and green for primary nucleation. (a) The concentrations at which the different reaction processes are 50% saturated. At these concentrations, saturation effects reduce the overall rates by 50%. (b) The fractional occupation of the catalytic surface for each reaction process over the range of monomer concentrations studied here. This makes a suitable definition for the degree of saturation of each process. Secondary nucleation is essentially fully saturated over all concentrations of interest, whereas elongation is largely unsaturated. Primary nucleation is fully saturated at the higher end of the range of concentrations studied. Where the fractional occupation is small, dissociation dominates over binding; where it is large, binding dominates over dissociation.

We now apply our model to study the aggregation of Aβ40 peptides into amyloid fibrils, which is believed to be a key upstream event in the development of Alzheimer’s disease. In addition to the obligatory primary nucleation and elongation processes, the aggregation reaction has been shown to depend critically on secondary nucleation.^10^ In the case of Aβ40, this process is known to undergo saturation at a monomer concentration of c. 6 *µ*M, at pH 7.4, and 37 °C. It was also suspected to undergo saturation in filament elongation at a higher concentration,^10^ since catalytic secondary nucleation alone could not describe the lack of concentration dependence of the aggregation rate at the highest concentrations studied [Fig. 3(a)]. However, this could not be verified, since the kinetic models available at the time could describe either catalytic secondary nucleation or catalytic elongation, but not both simultaneously. Equation (10a), however, permits this kind of analysis and allows simultaneous determination of *K*_*E*_, *K*_*P*_, and *K*_*S*_ from kinetic curves for filament formation from a wide range of initial monomer concentrations. Although catalytic elongation and secondary nucleation together offer a reasonable description of the kinetics [Fig. 3(b)], the data are in fact better described by catalytic primary and secondary nucleation, without saturation in elongation [Fig. 3(c)]. Additional support for this conclusion comes from a re-examining of the highly seeded aggregation experiments from Ref. 10. The initial rates of filament formation under highly seeded conditions are dominated by the elongation process.^16^ The linear dependence of these rates on monomer concentration indicates the absence of significant saturation effects in the elongation process at the concentrations studied (Fig. 4). Therefore, all data indicate that a saturation of primary nucleation, in addition to a saturation of secondary nucleation, occurs in the aggregation of Aβ40 at high micromolar concentrations (Fig. 5). This finding provides further insight into the molecular details of the nucleation mechanism responsible for the formation of the first aggregates directly from the monomer; since homogeneous primary nucleation cannot display saturation effects, the primary nucleation process undergone by Aβ40 *in vitro* must be a heterogeneous process in order to saturate. Heterogeneous nucleation is indeed much more common than its homogeneous counterpart for a wide range of nucleation phenomena, from simulations of hard sphere interactions to water condensation on dust particles.^32–36^ In the context of the highly purified Aβ40 samples studied in this work, nucleation most likely occurs at an interface such as the air-water interface or the surface of the reaction vessel. For some amyloid forming proteins, the effect of surface nucleation is so significant that aggregation can be completely inhibited in the absence of an air-water interface.^37–39^ In the context of the study of disease-related amyloids, our findings highlight the importance that surfaces may play a role in determining the rates of primary nucleation and that this effect should be taken into account when drawing disease-related conclusions from *in vitro* measurements.

## III. CONCLUSIONS

We have derived a single unified analytical solution to the kinetics of amyloid filament formation in which all reaction processes may be multistep and catalytic in nature. This model encompasses a wide range of filamentous growth kinetics and is superior to all prior analytical solutions in terms of accuracy and generality for the description of unseeded aggregation kinetics.

It has been suspected for some time that primary nucleation in amyloid formation is heterogeneous. This study presents some of the first direct evidence that this is indeed the case. The use of our model to fit kinetic data on amyloid aggregation at high monomeric protein concentrations provides a route to confirming the heterogeneous nature of amyloid nucleation for other proteins in the future.

To date, reaction conditions have often been chosen carefully to avoid multiple saturation effects, as they were previously difficult to interpret. By providing the ability to analyze such multiple saturated data, our results open the field up to wider studies of the effects of temperature, pH, and other important reaction conditions on filamentous growth kinetics. This may permit a move toward studying amyloid formation in body fluids, where reaction conditions may promote saturation effects, and toward studying other aggregating systems previously deemed pathological from a modeling perspective. Finally, our model will facilitate thermodynamic studies of activation energies, which require data to be collected over a range of temperatures, often including those at which multiple saturation effects occur. Overall, this new model has the potential to be transformative in the field of amyloid aggregation kinetics.

## Figures and Tables

**FIG. 6.**
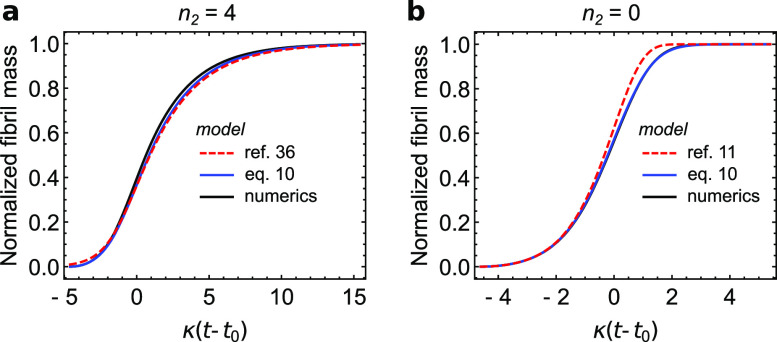
(a) The Richards solution (in blue) captures the dynamics of a system with secondary nucleation (*n*_2_ = 4) even more accurately than the Hamiltonian solution (in red). (b) The kinetics of fragmenting filament assembly are almost exactly captured by the new *c* = 3 Richards solution, significantly more accurately than the current standard; the Gompertz solution (in red) and the numerical solution (in black) to the moment equation for fragmenting filament assembly. *ε* = 0.01.

**FIG. 7.**
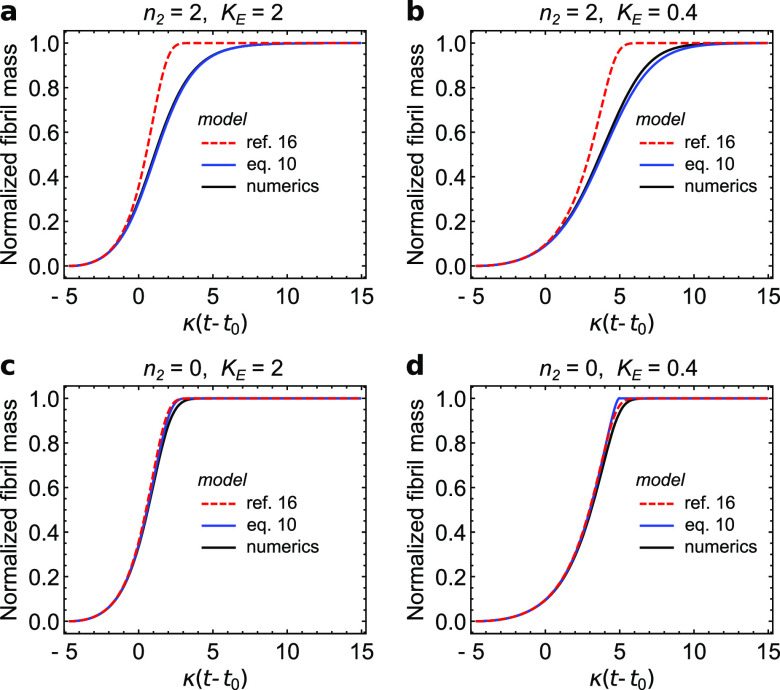
The kinetics of filament assembly with secondary nucleation and saturating elongation are almost exactly captured by Eq. (10a), which is a significant improvement upon an earlier analytical solution based on the Lambert-W function. However, the Richards solution offers little advantage over the Lambert solution for fragmenting systems. Black: numerical solution to the moment equations. Red: old (Lambert) analytical approximate solution. Blue: new (Richards) analytical approximate solution. *ε* = 0.01. (a) $n_{2}=2,\;K_{E}=2$. (b) $n_{2}=2,\;K_{E}=0.4$. (c) $n_{2}=0,\;K_{E}=2$. (d) $n_{2}=0,\;K_{E}=0.4$.

**FIG. 8.**
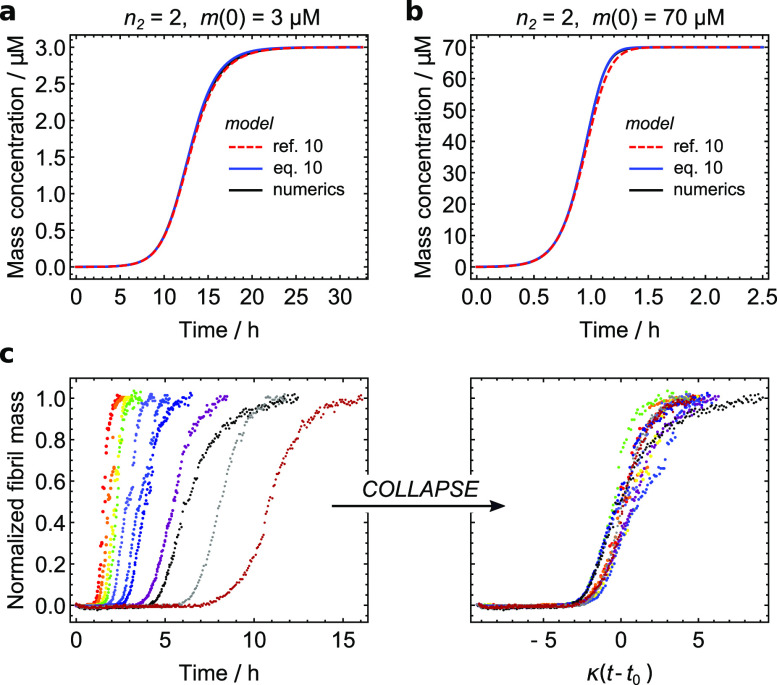
(a) and (b): using rate constants for Aβ40 aggregation taken from Ref. 10, we calculate kinetic curves for initial monomer concentrations of 3 *µ*M (a) and 70 *µ*M (b). The curve calculated according to the solution developed in this paper [blue, Eq. (10a)] matches the numerical solutions (black) almost precisely; even better than the already highly accurate analytical solution developed in Ref. 10 (red). (c) Raw Ab40 data from Ref. 10 at a range of concentrations 35–3.5 *μ*M (d) Scaling by *κ*′, and addition of $t_{0}=\ln\left(\varepsilon^{\prime }\right)/\kappa^{\prime }$, effectively collapses the data onto a universal curve, since experimental errors in these data are a greater source of variation from universality than the small differences in $n_{2}^{\prime }$ for each individual curve.

## APPENDIX A: REDUCING EQS. (8) TO A SINGLE DIMENSIONLESS EQUATION

Making use of the conservation-of-mass relation *m*(*t*) + *M*(*t*) = *m*_tot_, Eq. (8b) can be written as

$$\frac{1+\frac{m\left(t\right)}{K_{E}}}{m\left(t\right)}\frac{dM\left(t\right)}{dt}=-\left[\frac{1}{m\left(t\right)}+\frac{1}{K_{E}}\right]\frac{dm\left(t\right)}{dt}=2k_{+}P\left(t\right).$$ (A1)

Using *τ* = *κt*, this may be written as

$$-\left[\frac{d\;\log m\left(\tau \right)}{d\tau }+\frac{1}{K_{E}}\frac{dm\left(\tau \right)}{d\tau }\right]=\frac{2k_{+}}{\kappa }P\left(\tau \right).$$ (A2)

Differentiating with respect to *τ* and noting that *κdP*/*dτ* = *dP*/*dt*, we obtain

$$\begin{array}{ccc} & & -\left[\frac{d^{2}\;\log m\left(\tau \right)}{d\tau^{2}}+\frac{1}{K_{E}}\frac{d^{2}m\left(\tau \right)}{d\tau^{2}}\right]\\ & & \;=\frac{2k_{+}}{\kappa^{2}}\left(\frac{k_{n}m{\left(\tau \right)}^{n_{c}}}{1+{\left(\frac{m\left(\tau \right)}{K_{P}}\right)}^{n_{c}}}+\frac{k_{2}\;m{\left(\tau \right)}^{n_{2}}}{1+{\left(\frac{m\left(\tau \right)}{K_{S}}\right)}^{n_{2}}}M\left(\tau \right)\right), \end{array}$$ (A3)

and Eq. (9) is recovered by the substitution of *m*(*t*) = *m*_tot_*μ*(*t*) and *M*(*t*) = *m*_tot_(1 − *μ*(*t*)).

## APPENDIX B: DERIVATION OF GENERAL ANALYTICAL SOLUTION

To develop a perturbative solution to Eq. (9), we first define *ε* = *λ*^2^/2*κ*^2^ as our small parameter,

$$\mu \left(\tau \right)=\mu^{\left(0\right)}\left(\tau \right)+\varepsilon \;\mu^{\left(1\right)}\left(\tau \right)+\varepsilon^{2}\;\mu^{\left(2\right)}\left(\tau \right)+O\left(\varepsilon^{3}\right),$$ (B1)

where $O\left(\varepsilon^{3}\right)$ denotes a remainder term of the same order of magnitude as *ε*^3^. After solving the resulting equations separately for each order of *ε* and applying the initial conditions *μ*(0) = 1 and *μ*′(0) = 0, we find the following perturbation expansion:

$$\mu \left(\tau \right)=1-\varepsilon ae^{b\tau }+\frac{2+n_{2}^{\prime }}{3}\varepsilon^{2}a^{2}e^{2b\tau }+R,$$ (B2)

$$a=\frac{1+K_{S}}{K_{S}}\frac{K_{P}}{1+K_{P}},$$ (B3)

$$b=\sqrt{\frac{K_{E}}{1+K_{E}}\frac{K_{S}}{1+K_{S}}},$$ (B4)

$$n_{2}^{\prime }=n_{2}\frac{K_{S}^{n_{2}}}{1+K_{S}^{n_{2}}}-\frac{2}{1+K_{E}},$$ (B5)

where R denotes either terms of $O\left(\varepsilon^{3}\right)$ or terms that vanish in comparison to the dominant terms at each order in the limit *e*^*τ*^ ≫ 1. Since outside of this limit all terms of O(ε) and above can be neglected anyway, we may ignore R for the time being.

We see that the perturbative solution describes the early-time dynamics of Eqs. (8) as exponential growth. However, this formula is not accurate at long times, as it does not account for monomer depletion and therefore does not converge. To proceed, we note that this perturbation expansion has the same form as that given by the kinetics of noncatalytic biofilament self-assembly in Ref. 29 but with *ε* → *εa*, *τ* → *bτ*, and *n*_2_ → $n_{2}^{\prime }$. In that paper, the divergence was tamed by the use of dynamical renormalization group methods, yielding a remarkably accurate solution in the form of a Richards curve *y*(*x*) = 1 − (1 + *x*/*c*)^−*c*^, with *x* = *εe*^*τ*^, *y* = *μ*, and *c* = 3/(1 + 2*n*_2_). Given the identical functional form of the perturbative expansions for catalytic and noncatalytic biofilament self-assembly, the Richards solution may be immediately adopted here with appropriate replacements for *x* and *c*.

For small but nonvanishing values of *εa*, i.e., when most new filaments are formed through secondary processes, an expression of the above Richards form is expected to lose some accuracy at very early times, since the boundary conditions are met only in the limit *εa* → 0. However, since our perturbative expansion only corresponds to the Richards solution asymptotically in the limit *e*^*bτ*^ ≫ 1, we are free to modify our solutions so that they satisfy the boundary conditions *M*(0) = 0, as long as they recover their original form in this limit. The obvious choice is to replace *εae*^*bτ*^ with *εa*(*e*^*bτ*^ + *e*^−*bτ*^ − 2), which additionally ensures that the solution matches the first-order perturbative solution exactly. This yields Eq. (10) directly.

## APPENDIX C: COMPARISON TO PRIOR ANALYTICAL SOLUTIONS

### 1. The analytical solution is almost exact in the low-concentration limit

For initial monomer concentrations sufficiently far below the saturation concentrations and in the limit *e*^*τ*^ ≫ 1, Eq. (10a) recovers the universal single-step model for filamentous growth discovered in Ref. 29. We may investigate the solution error in this limit explicitly by comparing the ratio of the 3rd order terms of the perturbation expansions of the exact kinetics, *μ*^(3)^, and of the Richards curve, *L*^(3)^,

$$\frac{L^{\left(3\right)}}{\mu^{\left(3\right)}}=\frac{80+64n_{2}}{81+63n_{2}}.$$ (C1)

Not only is this remarkably close to 1, it is also exact for *n*_2_ = 1. We therefore expect this solution to be highly accurate. Indeed, in Fig. 6(a), we see that it is even more accurate for secondary nucleation than the highly accurate Hamiltonian solution given in Ref. 25.

To date,^11,25^ the kinetics of breakable filament assembly have been modeled with fairly high accuracy with a Gompertz curve, which is equal to the Richards solution presented here in the limit *c* → *∞*. However, our identification of *c* = 3/(1 + 2*n*_2_) suggests that this is not the best approximation possible. Instead, setting *n*_2_ = 0 gives *c* = 3. We discover that this Richards curve is indeed a superior approximation, converging on the exact kinetics almost precisely [Fig. 6(b)].

The Richards curve is traditionally used to model biological growth kinetics. More generally, the Richards curve describes autocatalytic growth kinetics for a system with a defined maximum product concentration. The parameter 1/*c* identifies the substrate dependence of the growth rate (here, the substrate is identified as monomer). The Gompertz solution, given by 1/*c* = 0, describes a system in which the substrate concentration does not limit the growth rate, and other factors, e.g., physical space limitations, give rise to a maximum product concentration. Viewed in this way, it is eminently reasonable that we do not use the Gompertz solution to describe fragmentation kinetics. Although the secondary process itself does not have substrate dependence (unlike secondary nucleation), the elongation process does, and aggregation should therefore still slow down as the monomer is depleted. The value given by our new approximation, 1/*c* = 1/3, may therefore be justified on conceptual and mathematical grounds.

### 2. Singly catalytic kinetics are captured with greater accuracy than earlier models

We now investigate the limit in which one of secondary nucleation or elongation may saturate but not both. Models have been previously derived for such singly saturating kinetics; we also compare our new solution to these models in this limit.

When only elongation may saturate, i.e., $K_{S},\;K_{P}\to \infty$, Eq. (10a) is usually highly accurate and found to be generally more accurate than the existing analytical solution based on the Lambert-W function (Fig. 7).

When only secondary nucleation may saturate, i.e., $K_{E},\;K_{P}\to \infty$, Eq. (10a) is almost an exact match to the numerical solutions of Eq. (8). The existing analytical solutions are highly accurate; however, Eq. (10a) still offers a small improvement in accuracy [Figs. 8(a) and 8(b)]. Moreover, the scaling of the solution is demonstrably correct since by scaling time by *κ*′ and offsetting it by *t*_0_ = ln(*ε*′)/*κ*′, the aggregation curves for A*β*40 at different starting concentrations almost fully collapse onto a single curve. A perfect collapse is prevented only by small variations in experimental conditions, for instance initial monomer concentration or sites for primary nucleation, and by each curve’s different value for *c*′ [Figs. 8(c) and 8(d)].

## APPENDIX D: EXTENSION TO THE CASE OF SLOW SECONDARY PROCESSES

For larger values of *ε* such that it can no longer be said that secondary processes dominate over primary nucleation for the production of new filaments, Eq. (10a) starts to break down at later times, since the secondary reaction order *n*_2_ no longer should control the late-time kinetics. To improve our solution, we can first investigate what form it should take in the limit *τ* → 0, i.e., when secondary processes are absent. We drop the final term in Eq. (9) and solve perturbatively, yielding (to second order in *ε*)

$$\begin{array}{ccc} \mu \left(\tau \right) & = & 1-\varepsilon \tau^{2}\frac{K_{E}}{1+K_{E}}\frac{K_{P}^{n_{c}}}{1+K_{P}^{n_{c}}}\\ & & +\;\varepsilon^{2}\tau^{4}\frac{K_{E}^{2}K_{P}^{2n_{c}}\left(3K_{E}+3K_{E}K_{P}^{n_{c}}+K_{P}^{n_{c}}n_{c}+K_{E}K_{P}^{n_{c}}n_{c}\right)}{6{\left(1+K_{E}\right)}^{3}{\left(1+K_{P}^{n_{c}}\right)}^{3}}. \end{array}$$ (D1)

We find that this matches our solution Eq. (10a) to the first order in *ετ*^2^ already; moreover, if we replace *c*′ with

$$c^{\prime }=\frac{3}{n_{c}^{\prime }},wheren_{c}^{\prime }=\frac{K_{P}^{n_{c}}}{1+K_{P}^{n_{c}}}n_{c}-\frac{3}{1+K_{E}},$$ (D2)

it is matched to the second order in *ετ*^2^ as well. We may compute the error in this approximation by comparing the third-order term of its expansion in *ετ*^2^ with that of the exact Oosawa solution in the unsaturated limit. The ratio of these terms, *r*_err_, is

$$r_{err}=\frac{15\left(1+n_{c}\right)+10n_{c}^{2}/3}{15\left(1+n_{c}\right)+4n_{c}^{2}}.$$ (D3)

We see that this is exact in the limit *n*_*c*_ = 0 and loses accuracy as *n*_*c*_ increases (with a limiting value of 5/6 as *n*_*c*_ → *∞*). We therefore expect that such a representation is accurate for values of *n*_*c*_ that are not too large, e.g., *n*_*c*_ < 4.

Of course, this is insufficient for describing intermediate values of *ε*′ and is not a single solution. By appropriate manipulation to obtain an effective $n_{2}^{\prime }$ in the limit of no secondary processes, $n_{2p}^{\prime }$, we can instead use a switching function for the secondary processes reaction order,

$$n_{2}^{†}=n_{2p}^{\prime }+\left(n_{2}^{\prime }-n_{2p}^{\prime }\right)\exp\left(-2\varepsilon^{\prime }\right),$$ (D4)

where

$$n_{2p}^{\prime }=\frac{1}{2}\left[n_{c}\frac{K_{P}^{n_{c}}}{1+K_{P}^{n_{c}}}-1-\frac{3}{1+K_{E}}\right].$$ (D5)

Using $n_{2}^{†}$ instead of $n_{2}^{\prime }$ in Eq. (10a) yields a fully universal solution valid for any possible kind of filamentous growth kinetics, single-step or catalytic multistep, and with or without secondary processes. Its only caveat is that it may not accurately describe filamentous growth under the rather specific circumstances of slow significant secondary processes, a high value of *n*_*c*_ > 3, and no saturation in primary nucleation.

## APPENDIX E: EXTENSION TO THE CASE OF SIMULTANEOUS FRAGMENTATION AND SECONDARY NUCLEATION

When a protein aggregation reaction that features secondary nucleation occurs under shaking conditions, both fragmentation and secondary nucleation may occur simultaneously. The same methods as employed in the rest of the paper may be employed to capture this behavior, yielding a modified expression for $k_{2}^{\prime }$ and $n_{2}^{\prime }$,

$$k_{2}^{\prime }=\frac{K_{S}^{n_{2}}+f\left(1+K_{S}^{n_{2}}\right)}{1+K_{S}^{n_{2}}},$$ (E1)

$$n_{2}^{\prime }=n_{2}\frac{K_{S}^{n_{2}}}{1+K_{S}^{n_{2}}}\frac{K_{S}^{n_{2}}}{K_{S}^{n_{2}}+f\left(1+K_{S}^{n_{2}}\right)}-\frac{2}{1+K_{E}},$$ (E2)

$$f=\frac{k_{-}}{k_{2}m_{tot}^{n_{2}}},$$ (E3)

where *k*_ is the rate constant for fibril fragmentation [and *k*_*M*(*t*) is the corresponding rate].

This approximation is reasonably accurate but less so than the other cases considered in this paper.
